# Association of plasma homocysteine with cardiovascular disease in American adults: a study based on the national health and nutrition examination survey database

**DOI:** 10.3389/fcvm.2025.1528540

**Published:** 2025-06-09

**Authors:** Zhaoming Huang, Qiaohui Zhang, Jie Zhang, Renyan Zhang, Yujie Huang, Demin Xu

**Affiliations:** ^1^ School of Public Health, Xiamen University, Xiamen, Fujian, China; ^2^ Zhongshan Hospital (Xiamen), Fudan University, Xiamen, Fujian, China

**Keywords:** cardiovascular disease, homocysteine, logistic regression, linear trend analysis, National Health and Examination Survey

## Abstract

**Objective:**

The purpose of this study was to investigate the correlation between plasma homocysteine (Hcy) levels and cardiovascular disease (CVD) in United States adults based on the National Health and Examination Survey (NHANES) database of the United States.

**Methods:**

Data from two survey periods (2003–2006) in the NHANES database were used as the research data set. Plasma Hcy levels are considered an independent variable, while CVD is a dependent variable. Weighted logistic regression, linear trend analysis, subgroup analysis and limiting cubic spline plots were used for analysis. A total of 4,418 samples were included.

**Results:**

In the weighted logistic regression model, a significant positive correlation between Hcy level and CVD risk was observed (*P* for trend = 0.007).The subgroup analysis revealed that various characteristics such as age, race, education level, obesity, alcohol use, diabetes, and hypertension did not affect this positive correlation (*P* for interaction ≥0.05). The nonlinear association between Hcy level and CVD risk was explored by limiting cubic spline plots, revealing the overall significant trend (*P* for overall <0.0001) and the significant nonlinear trend (*P* for nonlinear <0.01).

**Conclusion:**

In this large cross-sectional study, an increase in Hcy levels leads to an increased risk of CVD. There is a nonlinearly positive correlation between Hcy levels and the risk of CVD.

## 1 Introduction

Cardiovascular diseases (CVD) remain a major cause of death and disability globally, posing a significant threat to global health (1). According to the findings of the Global Burden of Disease (GBD) studies, the global incidence of cardiovascular diseases has grown by 93% over the past three decades, reaching an astonishing 523 million cases by 2019, which has imposed a heavy economic burden on global healthcare systems and communities (2).

In recent years, an increasing number of studies have shown that elevated levels of serum homocysteine (Hcy) are associated with atherosclerosis, and it is an independent risk factor for atherosclerosis (3). Additional research confirms that: Elevated serum homocysteine (Hcy) levels are an independent risk factor for the formation of atherosclerotic plaques. This factor is equally important as hypertension, hyperlipidemia, diabetes, and other risk factors, and can lead to an increase in the incidence and mortality of cardiovascular and cerebrovascular diseases (4).

Therefore, reducing Hcy levels may lower the risk of CVD, with significant preventive implications. However, there is currently a lack of large-scale population studies specifically focused on the association between Hcy and CVD risk.

We comprehensively analyzed the relationship between Hcy levels and CVD in American adults using data from two cycles of the National Health and Nutrition Examination Survey (NHANES) from 2003 to 2006. This analysis also aimed to uncover whether the potential relationship between Hcy and CVD is more pronounced in specific populations. These findings provide strong support for further investigations into the pathogenesis of CVD and the relevance of Hcy levels as a molecular marker in CVD patients.

## 2 Materials and methods

### 2.1 Study population

The data source for this study is based on the NHANES database (http://www.cdc.gov/nchs/nhanes.htm), which originates from a stratified, multi-stage study that combines interviews, physical examinations, and laboratory tests to assess the health and nutritional status of the US population, conducted by the National Center for Health Statistics. This freely accessible database has been approved by the Institutional Review Board of the National Center for Health Statistics and has obtained informed consent from all participants. The procedures follow the principles of the Helsinki Declaration. The NHANES data used in this study is publicly available; therefore, this study is considered not to require ethical or administrative approval.

### 2.2 Selection criteria

**Figure 1:**
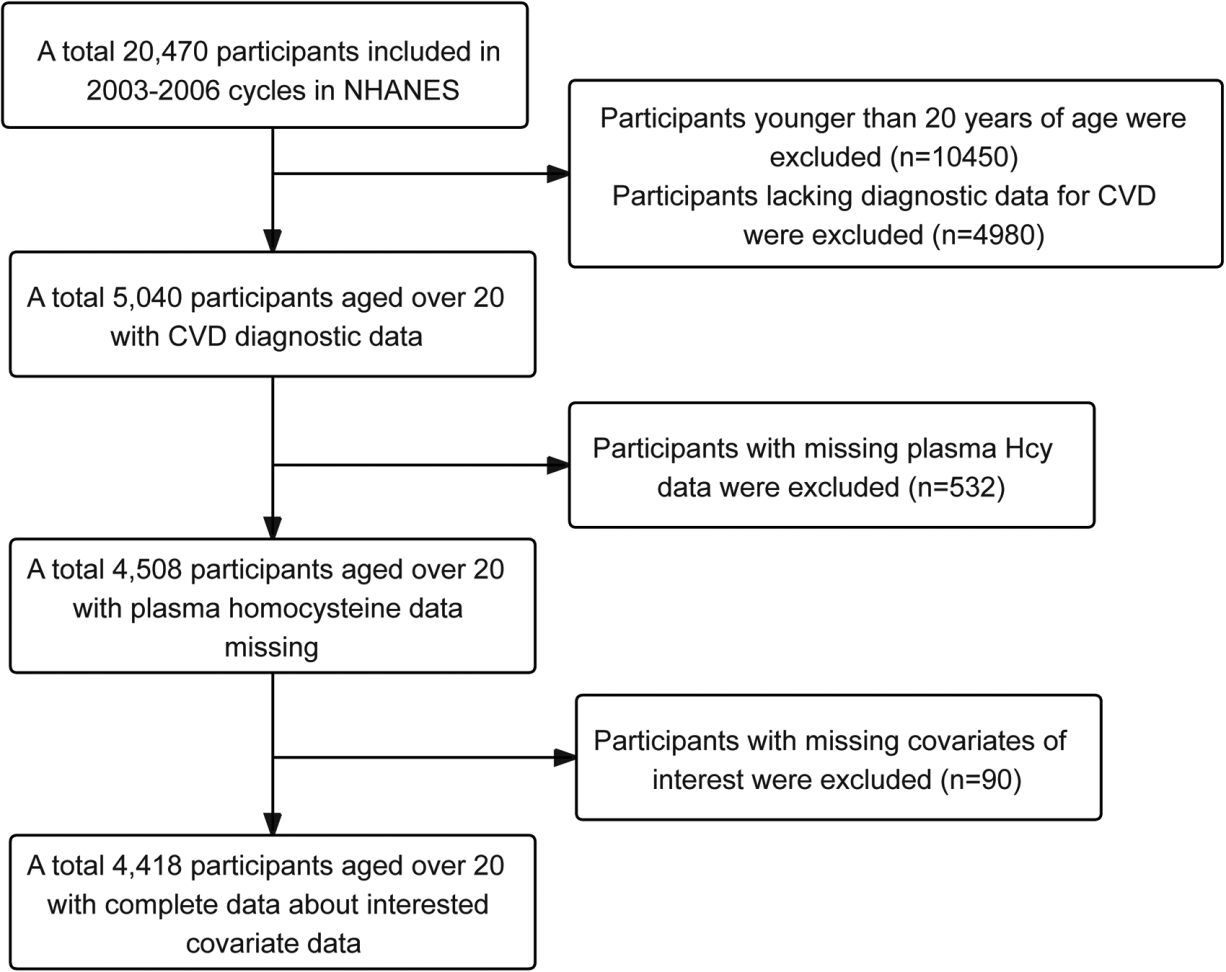
Flowchart illustrating the sample selection process for NHANES 2003 to 2006.

In this study, information was collected from 20,470 participants across two consecutive cycles (2003–2006) of the NHANES database. Given that CVD primarily affects adults, and with a lower prevalence in the under-20 age group, 10,450 participants under 20 years of age were excluded. Participants lacking CVD diagnostic data, based on the CVD questionnaire, were also excluded, amounting to 4,980 individuals. Furthermore, 532 participants without plasma Hcy data and 90 participants lacking relevant covariate information were excluded. Ultimately, 4,418 participants met the criteria and were included in the study. The detailed process of participant selection is illustrated in Figure 1.

### 2.3 Measurement of plasma Hcy

Total homocysteine (Hcy) in plasma is measured using the “Abbott HCY assay”, an automated fluorescence polarization immunoassay (FPIA) from Abbott Diagnostics (5).

### 2.4 Main outcomes

The outcome of this study was considered to the incident of CVD. The definition of CVD is based on self-reported issues: Ever told you had congestive heart failure, coronary heart disease, angina/angina pectoris, heart attack or stroke. Respondents who answer “yes” to these questions are defined as having CVD (6, 7).

### 2.5 Covariates

Age is divided into three groups: 20–39, 40–59, and ≥60 years. Gender is divided into male and female. Race is divided into Mexican Americans, non-Hispanic whites, non-Hispanic blacks, and other. Marital status is divided into married (married or living with a partner) or unmarried (other). Poverty status is divided into poor and not poor: The poverty income ratio (PIR) ≥ 1 is defined as the non-poor, and PIR < 1 is defined as the poor. Education level is divided into below high school and high school or above. obesity is divided into Yes and No: obesity is defined as BMI ≥ 30, and non-obesity as BMI < 30 (8) [Body Mass Index (BMI): BMI is calculated as weight (kg) divided by height squared (m^2^)].

Smoking is categorized as Yes or No: smoking are those who answer “every day” or “some days” to the question “Do you smoke now?” and also answer “yes” to the question “Have you smoked at least 100 cigarettes in your life?”; the rest are classified as not smoking. Alcohol use was determined after the response to the question “Had at least 12 alcohol drinks/1yr?” with “Yes”or “No” (1 drink refers to 12 ounces of beer, 4 ounces of wine, or 1 ounce of liquor) (9).

Hypertension is defined as meeting any of the following criteria: (1) an average systolic/diastolic blood pressure of ≥140/90 mmHg, (2) a previous diagnosis by a doctor or healthcare professional, or (3) currently taking antihypertensive medication (10, 11). Diabetes is defined as meeting any of the following criteria: (1) being told by a doctor they have diabetes, (2) taking antidiabetic medication, (3) having glycated hemoglobin >6.5%, or (4) fasting blood glucose >126 mg/100 ml (12).

### 2.6 Statistical analysis

In this study, the “tableone” package was used to generate baseline tables. Participants were grouped according to whether they had CVD or not, and based on this, sample size and proportion of categorical variables were calculated, as well as mean and standard deviation. The sample size (*n*) was unweighted, whereas proportions [*n* (%)], means, and SD were adjusted according to weights. One-way ANOVA and chi-square tests were used to compare continuous and categorical variables, respectively.

Divide the plasma Hcy levels into quartiles. If the *P* < 0.05 in univariate analysis, input the variable into a multivariate logistic regression model. Weighted multivariate logistic regression analysis is used to estimate the odds ratio (OR) and 95% confidence interval (95% CI) for the association between CVD and Hcy, and a linear trend analysis is performed further, with *P* < 0.05 considered statistically significant.

The models in this study include Model 1 (Adjusted for gender, age, race, marital status, education level, and poverty status.); Model 2 (Adjusted for gender, age, race, marital status, education level, poverty status, obesity, smoking, and alcohol use.); and Model 3 (Adjusted for gender, age, race, marital status, education level, poverty status, obesity, smoking, alcohol use, hypertension, and DM.). Restricted cubic splines (RCS) are used in the three models to explore the association between Hcy levels and the prevalence of CVD. Subgroup analyses are conducted on the relationship between Hcy and CVD, stratified by age, race, education level, obesity, alcohol use, hypertension, and DM, to assess potential modifying factors. The data are weighted using complex survey sampling analysis methods to ensure they are representative of the US population. A two-sided *p*-value of <0.05 is considered statistically significant. All statistical analyses were performed using R (version 4.4.1).

## 3 Results

### 3.1 Characteristics of the study population

Table 1 provides a brief summary of the baseline characteristics distribution of the study population. The study included a total of 4,418 people, of which 582 were patients with CVD, and 3,836 were non-CVD patients. The sample population comprised 48.3% men and 51.7% women. The proportions of young patients (20–40 years), middle-aged patients (40–59 years) and older adults (≥60 years) were 38.6%, 38.8% and 22.6%, respectively. Of the participants, 36.9% had hypertension and 10.3% had a history of diabetes.

**Table 1:** Characteristics distribution of CVD patients and non-CVD patients.

Characteristics	Overall (*n* = 4,418)	Non-CVD (*n* = 3,836)	CVD (*n* = 582)	*P*
Age				<0.001
20–39	1538 (38.6%)	1521 (42.2%)	17 (4.8%)	
40–59	1256 (38.8%)	1156 (39.8%)	100 (30.0%)	
≥60	1624 (22.6%)	1159 (18.0%)	465 (65.2%)	
Gender				0.677
Male	2137 (48.3%)	1827 (48.2%)	310 (49.3%)	
Female	2281 (51.7%)	2009 (51.8%)	272 (50.7%)	
Race				0.04
Mexican American	884 (7.7%)	795 (8.2%)	89 (3.4%)	
Non-Hispanic White	2363 (72.6%)	1983 (71.8%)	380 (80.2%)	
Non-Hispanic Black	846 (10.8%)	767 (11.0%)	79 (8.9%)	
Other	325 (8.9%)	291 (9.1%)	34 (7.5%)	
Marital status				0.055
Married	2694 (64.6%)	2357 (65.0%)	337 (60.3%)	
Unmarried	1724 (35.4%)	1479 (35.0%)	245 (39.7%)	
Education level				<0.001
Below high school	1279 (17.8%)	1042 (16.5%)	237 (30.0%)	
High School or above	3139 (82.2%)	2794 (83.5%)	345 (70.0%)	
Poverty status				0.352
Poor	763 (11.9%)	662 (11.8%)	101 (13.2%)	
Not poor	3655 (88.1%)	3174 (88.2%)	481 (86.8%)	
Obesity				0.005
Yes	1458 (32.2%)	1233 (31.3%)	225 (40.1%)	
No	2960 (67.8%)	2603 (68.7%)	357 (59.9%)	
Smoking				0.072
Yes	992 (25.3%)	898 (25.7%)	94 (20.8%)	
No	3426 (74.7%)	2938 (74.3%)	488 (79.2%)	
Alcohol use				0.003
Yes	2827 (66.7%)	2489 (67.8%)	338 (57.0%)	
No	1591 (33.3%)	1347 (32.2%)	244 (43.0%)	
Hypertension				<0.001
Yes	1900 (36.9%)	1453 (32.9%)	447 (74.2%)	
No	2518 (63.1%)	2383 (67.1%)	135 (25.8%)	
DM				<0.001
Yes	603 (10.3%)	416 (8.2%)	187 (30.1%)	
No	3815 (89.7%)	3420 (91.8%)	395(69.9%)	
Hcy (μmol/L)	9.13 (4.28)	8.86 (3.98)	11.61 (5.89)	<0.001

Categorical variables are presented as *n* (%), while continuous variables are presented as mean (sd); *n* is unweighted, *n* (%), mean, and sd are weighted.

DM, diabetes; Hcy, homocysteine.

Divide participants into two groups based on whether they have CVD. There were statistically significant differences (*P* < 0.05) between the two groups in terms of age, race, education level, obesity, alcohol use, hypertension, diabetes, and homocysteine levels. However, there were no statistically significant differences in terms of gender, marital status, poverty status and smoking (*P* > 0.05).

Compared to those without CVD, participants with CVD were more likely to be older, non-Hispanic White, less educated, obesity and not alcohol use. Furthermore, they were more likely to have a history of hypertension and diabetes. There was no difference in smoking distribution, but CVD patients were more likely to have higher levels of homocysteine.

### 3.2 Association between Hcy and CVD

As shown in Table 2, three models were constructed in the population. Taking Hcy level as a continuous variable, the risk of CVD increased with every 1SD increase in Hcy level in all three models (*P* < 0.05). In models 1, 2, and 3, the corresponding OR (95% CI) for each 1SD increase in Hcy levels was 1.25 (1.11, 1.40), 1.25 (1.11, 1.42), and 1.19 (1.05, 1.35), respectively. If the Hcy level is categorized into quartile variables, with the first quartile (Q1) as the reference, weighted logistic regression analysis and linear trend analysis were performed. According to the results of the linear trend analysis, there is a significant positive correlation between Hcy level and the risk of CVD in all three models (*P* < 0.05). Further analysis of the impact of Hcy levels on the occurrence of CVD showed that compared to the first quartile (Q1), the fourth quartile (Q4) of Hcy levels significantly increased the risk of CVD in models 1 and 2 (*P* < 0.05), while there was no significant relationship in model 3 (*P* > 0.05).

**Table 2:** Relationship model between serum Hcy levels and CVD risk adjusted for different confounding factors.

Variable	Model 1 OR (95% CI)	*P*	Model 2 OR (95% CI)	*P*	Model 3 OR (95% CI)	*P*
Hcy continuous	1.25 (1.11, 1.40)	0.002	1.25 (1.11, 1.42)	0.004	1.19 (1.05, 1.35)	0.017
Hcy quartiles						
Q1 (<6.92)	Ref.		Ref.		Ref.	
Q2 (6.92–8.32)	1.34 (0.74, 2.44)	0.274	1.32 (0.66, 2.62)	0.326	1.30 (0.43, 3.90)	0.415
Q3 (8.32–10.24)	1.48 (0.92, 2.36)	0.088	1.46 (0.85, 2.52)	0.126	1.44 (0.61, 3.43)	0.209
Q4 (≥10.24)	2.66 (1.59, 4.46)	0.003	2.60 (1.46, 4.66)	0.01	2.28 (0.96, 5.38)	0.054
*P* for trend	0.001		0.001		0.007	

Model 1: Adjusted for gender, age, race, marital status, education level, and poverty status.

Model 2: Adjusted for gender, age, race, marital status, education level, poverty status, obesity, smoking, and alcohol use.

Model 3: Adjusted for gender, age, race, marital status, education level, poverty status, obesity, smoking, alcohol use, hypertension, and DM.

DM, diabetes; Hcy, homocysteine.

**Figure 2:**
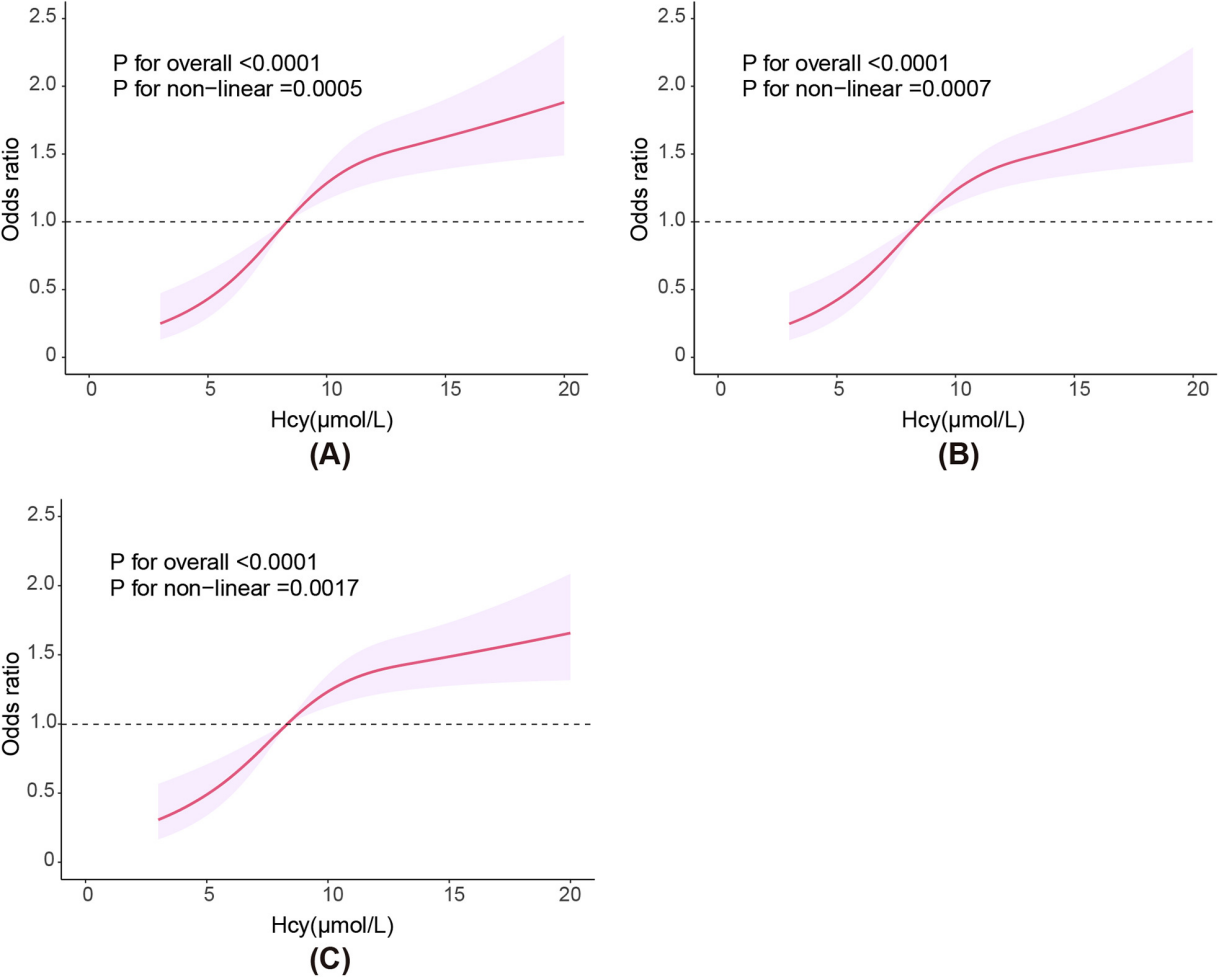
RCS plot showing the association between plasma Hcy level and risk of CVD. **(A)** Model 1, **(B)** Model 2, **(C)** Model 3. Model 1: Adjusted for gender, age, race, marital status, education level, and poverty status. Model 2: Adjusted for gender, age, race, marital status, education level, poverty status, obesity, smoking, and alcohol use. Model 3: Adjusted for gender, age, race, marital status, education level, poverty status, obesity, smoking, alcohol use, hypertension, and DM. DM, diabetes; Hcy, homocysteine.

Figure 2 illustrates the non-linear association between Hcy levels and CVD risk. The results of the RCS curves in all three models indicate a significant overall trend between Hcy levels and CVD risk (*P* for overall <0.0001). A significant non-linear association was observed in all three models (*P* for nonlinear <0.01). Risk of CVD continuously increased with higher Hcy levels.The RCS curves show a consistent overall trend between Hcy levels and CVD risk across all three models, indication a stable association between the two.

### 3.3 Stratification analysis

**Figure 3:**
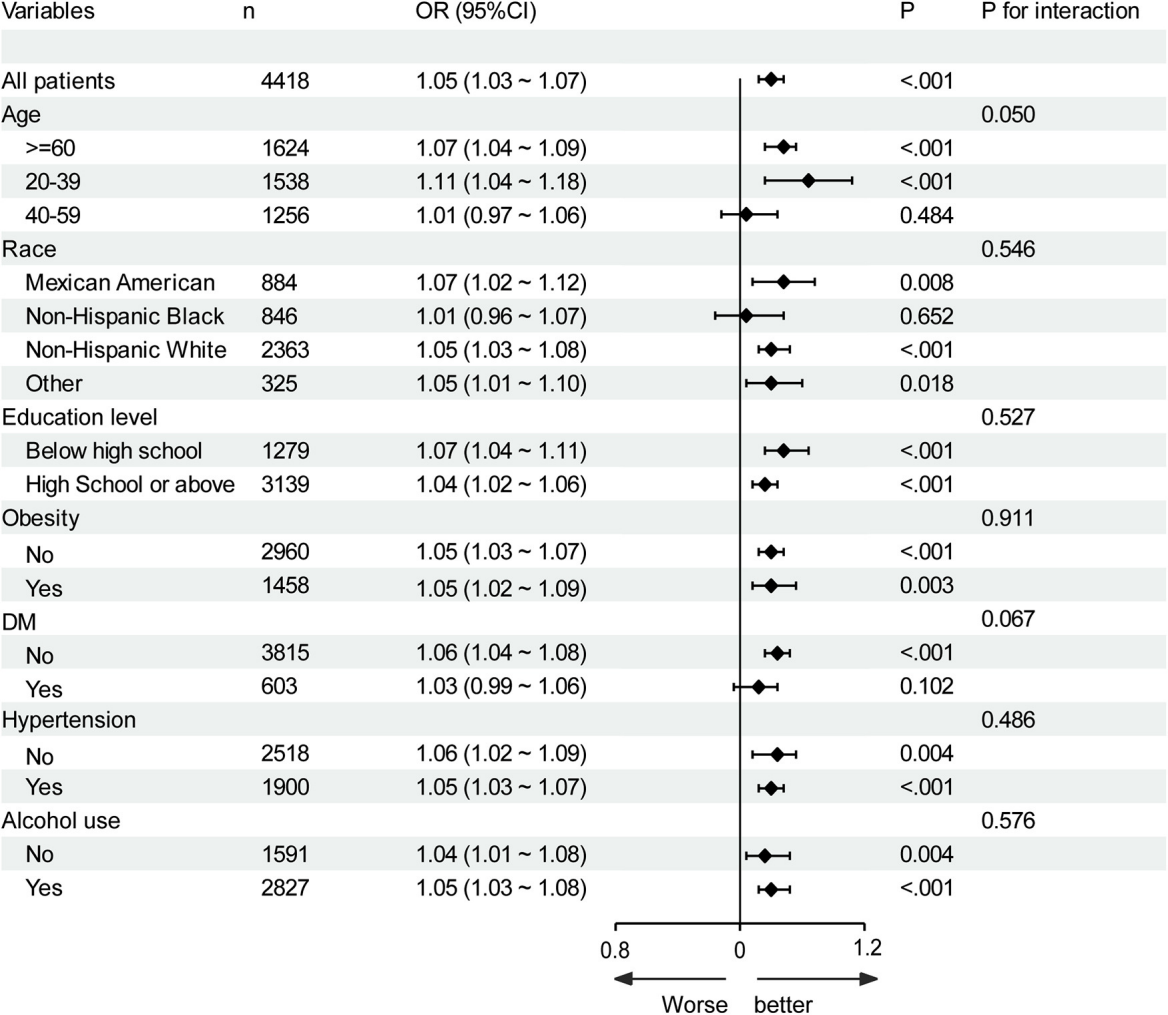
Subgroup analysis of the association of homocysteine with CVD. Results were adjusted for all covariates except the corresponding stratification variable. DM, diabetes.

Subgroup analysis was performed based on age, race, education level, obesity, alcohol use, diabetes, and hypertension (Figure 3). Hcy was significantly associated with CVD in all subgroups. No interaction effects were observed between plasma Hcy concentration and age (*P* for interaction = 0.05), race (*P* for interaction = 0.546), education level (*P* for interaction = 0.527), obesity (*P* for interaction = 0.911), alcohol use (*P* for interaction = 0.576), diabetes (*P* for interaction = 0.067), and hypertension (*P* for interaction = 0.486); therefore, these variables did not significantly alter the association between Hcy and CVD. Although there was no significant association between Hcy and CVD in the 40–59 age group, non-Hispanic black population, and diabetes group, there was no interaction effect (all *P* for interaction ≥ 0.05).

## 4 Discussion

We selected data from the nationally representative NHANES database (2003–2006) to assess the association between Hcy levels and the risk of CVD. Our study found that higher Hcy levels are associated with an increased risk of CVD, showing a significant positive correlation between Hcy levels and CVD risk. Further analysis showed that there was a significant nonlinear positive correlation between Hcy level and CVD risk. This discovery may play a certain role in the diagnosis and prevention of cardiovascular diseases.

Hcy can lead to dysfunction and even apoptosis of vascular endothelial cells, which is one of the important initiating factors in the development of artery atherosclerosis. High levels of Hcy reduce the expression of endothelial nitric oxide synthase (eNOS), leading to a decrease in endogenous nitric oxide (NO) and an increase in the production of malonaldehyde (MDA) (13). This results in a reduction, and potentially a complete loss, of vasodilatory function, accelerating the progression of atherosclerotic lesions (14). Moreover, Hcy can also induce endothelial cell apoptosis through activation of the c-Jun N-terminal kinase (JNK) (15, 16).

High concentration of Hcy can lead to the migration and proliferation of vascular smooth muscle cells (VSMCs) through multiple pathways. These pathways include enhancing the expression of caveolin to inhibit the action of eNOS and NO (17), enhancing the expression of key proteins and molecules in the phosphatidylinositol 3-kinase/protein kinase B (PI3K/Akt) signaling pathway (18), and inducing VSMCs to secrete matrix metalloproteinase-2 (MMP2), affecting the dynamic balance of extracellular matrix in and around cells (19). These mechanisms collectively promote the migration and proliferation of VSMCs, thereby intensifying the process of atherosclerosis in coronary arteries.

Oxidative stress is one of the important pathogenic mechanisms in the development of artery atherosclerosis. The metabolism of Hcy produces thiols that can auto-oxidize into active oxygen clusters such as hydrogen peroxide, leading to an increase in the content of oxidants in the body (20). At the same time, Hcy inhibits the expression and secretion of extracellular superoxide dismutase (SOD), further triggering oxidative stress. This state of oxidative stress disrupts the balance of the oxidation-antioxidation system within the body, causing lipid peroxidation, structural and functional changes in proteins, and DNA damage, thus promoting the development of atherosclerosis (21–23).

Inflammatory factors play a crucial role in the occurrence and development of atherosclerosis. Hcy can enhance the oxidative stress of vascular endothelial cells, leading to the production of a series of reactive oxygen species (ROS) in the body. An excess of ROS can oxidize low-density lipoprotein (LDL) into oxidized LDL, which is cytotoxic and can cause the destruction of fibroblast-actin microfilaments, leading to a disordered distribution. This results in an increase in the permeability of endothelial cells (24). Consequently, this causes a large expression of inflammatory factors such as vascular cell adhesion molecule-1 (VCAM-1) and *P*-selectin, making mononuclear macrophages more prone to enter the endothelial cells through adhesion molecules, exacerbating cellular damage and further promoting the occurrence and development of atherosclerosis (25).

The levels of Hcy show a clear relationship with the development of atherosclerosis, suggesting that Hcy may exert its effect on CVD through the aforementioned mechanisms.

Some research indicates that Hcy is an independent risk factor for cardiovascular diseases, with elevated serum Hcy levels being associated with CVD events (26–29).

In a prospective study by Esteghamati et al. over an 8.5-year follow-up on two sub-cohorts, it was shown that Hcy is associated with the development of metabolic syndrome, and both Hcy and metabolic syndrome are independent risk factors for coronary heart disease (CHD) (30). A cross-sectional study by Mohamed et al. involving 972 non-diabetic patients showed significant associations between high homocysteine levels (*P* = 0.01) and smoking (*P* = 0.004) and an increase in the carotid total plaque area (TPA) (31). TPA is a strong predictor of cardiovascular risk, measured through carotid ultrasound (32).

A meta-analysis found that after adjusting for other known risk factors, a 25% increase in total homocysteine (Hcy) in the serum (approximately 3 μmol/L) was associated with a 10% increase in the risk of cardiovascular events and a 20% increase in the risk of stroke (33). Another systematic review and meta-analysis, based on 26 articles, found that after adjusting for traditional CHD risk factors, for every 5 μmol/L increase in Hcy levels, the risk of CHD events increased by about 20% (34).

A systematic review and meta-analysis incorporating 35 studies that met the design criteria showed that in most of the studies, an increase in carotid intima-media thickness was observed when homocysteine levels were above normal. It was concluded that homocysteine is highly associated with atherosclerotic cardiovascular disease in young and overweight patients. This meta-analysis focused on the role of homocysteine in the formation of atherosclerotic cardiovascular disease in young and pediatric patients (35).

In addition, several studies have explored the association between Hcy and CVD events. Veeranna et al. conducted a multi-ethnic study on atherosclerosis and found that in the NHANES III database, Hcy level (>15 μmol/L) significantly predicted CVD (adjusted hazard ratio [aHR]: 1.79, 95% confidence intervals [CI]: 1.19–1.95; *P* = 0.006) and CHD events (aHR: 2.22, 95% CI: 1.20–4.09; *P* = 0.01) in the MESA trial and CVD (aHR: 2.72, 95% CI: 2.01–3.68; *P* < 0.001) and CHD mortality (aHR: 2.61, 95% CI: 1.83–3.73; *P* < 0.001) in the NHANES III (36). Drewes et al. concluded from a prospective study on pravastatin in high-risk elderly individuals (a double-blind, randomized, placebo-controlled trial with a follow-up of 3.2 years) that patients with higher Hcy levels had a higher risk of fatal and non-fatal CHD compared to those with lower Hcy levels (adjusted OR: 1.8, 95% CI: 1.2–2.5, *P* = 0.001) (37).

In this study, Hcy levels were significantly positively correlated with the risk of CVD in all three models. In model 3, which adjusted for all confounding factors, this correlation remained stable (*P* for trend = 0.007). Moreover, in model 3, a clear non-linear positive correlation was also observed (*P* for overall < 0.0001, *P* for nonlinear = 0.0017), similar to the above results.

Despite the significant results obtained in our research, it is important to acknowledge some limitations. Firstly, NHANES was designed as a cross-sectional study, inherently observational; thus, it does not allow for the establishment of causality and cannot fully rule out residual confounding. Future longitudinal studies are needed to ascertain the predictive value of Hcy for CVD risk. Secondly, self-reporting of CVD in the questionnaire may introduce bias. However, the questionnaires administered in this study have been widely used in previous studies to assess cardiovascular disease (6, 7). Thirdly, dietary habits, particularly nutraceutical intake (e.g., folate, vitamin B6/B12, and omega-3 fatty acids), may confound or modify the Hcy-CVD relationship by altering Hcy levels or lipid metabolism (38). This study did not include detailed dietary intake and nutraceutical usage records, limiting our ability to analyze interactions between these factors and Hcy/CVD risk. Future studies should incorporate dietary metrics, such as Mediterranean Diet scores and supplement use, to clarify these interactions.

Fourth, our analysis relied on a single Hcy measurement, which may not fully capture intra-individual variability over time. Longitudinal studies with repeated Hcy assessments are warranted to validate our findings. Fifth, the use of older NHANES data (2003–2006) limits generalizability to modern populations. Future research should use new cohorts to validate the results. Sixth, the absence of detailed pharmacological and dietary data hinders exploration of drug-diet-Hcy interactions.

Lastly, CVD has many potential influencing factors, and although we included as many relevant covariates as possible in our model, we cannot completely exclude the effects of unmeasured or other confounding factors, such as dietary patterns, physical activity, genetic predisposition, and environmental exposures that may influence both Hcy levels and CVD risk. Future studies incorporating these variables are warranted to refine the association between Hcy and CVD. Despite these limitations, our research still demonstrates a positive correlation between Hcy levels and CVD risk.

Future research should prioritize prospective cohorts with serial Hcy and B-vitamins measurements, stratified analyses by demographic and metabolic subgroups, and randomized clinical trials testing Hcy-lowering therapies to confirm causality and identify high-risk populations.

## 5 Conclusion

In this large cross-sectional study, an increase in Hcy levels leads to an increased risk of CVD. There is a positive correlation between Hcy levels and the risk of CVD. Further analysis revealed that the relationship between Hcy levels and CVD risk is nonlinearly positive.

## Data Availability

The raw data supporting the conclusions of this article will be made available by the authors, without undue reservation.
