# Supplementary material for: Association of plasma homocysteine with cardiovascular disease in American adults: a study based on the national health and nutrition examination survey database
Source: Front Cardiovasc Med. 2025 Jun 9;12:1528540. doi: 10.3389/fcvm.2025.1528540 (PMC12183184; doi:10.3389/fcvm.2025.1528540)
Supplement: Supplementary file 3 [file Table1.docx]

Supplementary Material

# Supplementary Tables

Supplementary Table1 Covariate grouping and definition in this study

| Variables | Group | Explanation |
| --- | --- | --- |
| Age | 20-39 | None |
|  | 40-59 |  |
|  | >=60 |  |
| Gender | Male | None |
|  | Female |  |
| Race | Mexican American | None |
|  | Non-Hispanic White |  |
|  | Non-Hispanic Black |  |
|  | Other |  |
| Marital status | Yes | married (married or living with a partner) or unmarried (other) |
|  | No |  |
| Education level | Below high school | None |
|  | High School or above |  |
| Poverty status | Poor | The poverty income ratio (PIR)≥1 is defined as the non-poor, and PIR<1 is defined as the poor. |
|  | Not poor |  |
| Obesity | Yes | obesity is defined as BMI≥30, and non-obesity as BMI<30 (Body Mass Index (BMI): BMI is calculated as weight (kg) divided by height squared (m2)) |
|  | No |  |
| Smoking | Yes | smoking are those who answer “every day” or "some days" to the question “Do you smoke now?” and also answer “yes” to the question “Have you smoked at least 100 cigarettes in your life?”; the rest are classified as not smoking |
|  | No |  |
| Alcohol use | Yes | Alcohol use was determined after the response to the question “Had at least 12 alcohol drinks/1 yr?” with “Yes”or “No” (1 drink refers to 12 ounces of beer, 4 ounces of wine, or 1 ounce of liquor) |
|  | No |  |
| Hypertension | Yes | meeting any of the following criteria: (1) an average systolic/diastolic blood pressure of ≥140/90 mmHg, (2) a previous diagnosis by a doctor or healthcare professional, or (3) currently taking antihypertensive medication |
|  | No |  |
| DM | Yes | meeting any of the following criteria: (1) being told by a doctor they have diabetes, (2) taking antidiabetic medication, (3) having glycated hemoglobin >6.5%, or (4) fasting blood glucose >126 mg/100 mL |
|  | No |  |

DM= diabetes

Supplementary Table2 Weighted multivariate logistic regression analysis of the association between Hcy and CVD

| Variables | OR（95%CI） | *P* |
| --- | --- | --- |
| Hcy |  |  |
| Q1 | Ref. |  |
| Q2 | 1.30 [0.43, 3.90] | 0.415 |
| Q3 | 1.44 [0.61, 3.43] | 0.209 |
| Q4 | 2.28 [0.96, 5.38] | 0.054 |
| Age |  |  |
| 20-39 | Ref. |  |
| 40-59 | 4.08 [1.21, 13.80] | 0.038 |
| >=60 | 11.75 [3.35, 41.15] | 0.014 |
| Race |  |  |
| Mexican American | Ref. |  |
| Non-Hispanic White | 1.87 [0.82, 4.23] | 0.082 |
| Non-Hispanic Black | 1.25 [0.56, 2.80] | 0.357 |
| Other | 1.68 [0.20, 14.35] | 0.406 |
| Education |  |  |
| Below high school | Ref. |  |
| High School or above | 0.59 [0.37, 0.93] | 0.038 |
| Obesity |  |  |
| No | Ref. |  |
| Yes | 1.18 [0.72, 1.95] | 0.289 |
| Alcohol use |  |  |
| No | Ref. |  |
| Yes | 0.88 [0.48, 1.62] | 0.462 |
| DM |  |  |
| No | Ref. |  |
| Yes | 2.13 [1.18, 3.85] | 0.032 |
| Hypertension |  |  |
| No | Ref. |  |
| Yes | 2.15 [1.16, 4.00] | 0.033 |

Hcy= homocysteine; DM= diabetes

# Supplementary Figures

**
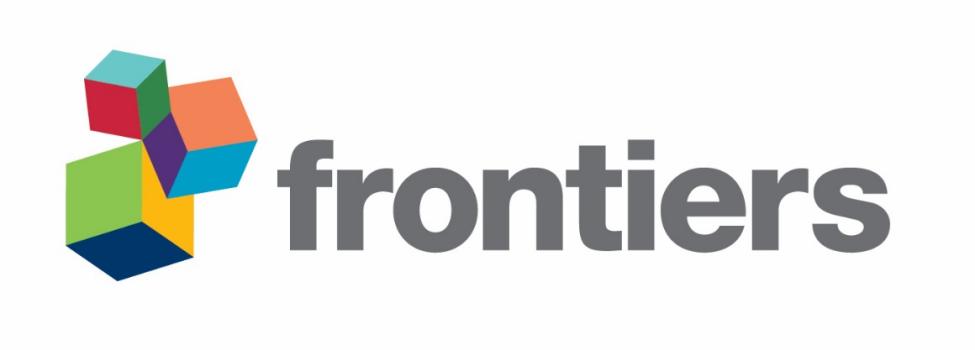
**
